# Bacteriophage as an anti-biofilm agent against *Pseudomonas aeruginosa* from wound infection

**DOI:** 10.1371/journal.pone.0334139

**Published:** 2025-10-09

**Authors:** Padma Shrestha, Sujit Tandukar, Manisha Shrestha, Sanjit Shrestha, Anup Subedee, Jivan Shakya, Reshma Tuladhar

**Affiliations:** 1 Institute for Research in Science and Technology, Lalitpur, Nepal; 2 Central Department of Microbiology, Tribhuvan University, Kirtipur, Nepal; 3 Pathology Department, Kirtipur Hospital, Nepal; 4 Department of Internal Medicine, Public Health Concern Trust (PHeCT)-Nepal Kirtipur Hospital,; 5 Mycobacterial Research Laboratories (MRL), Anandaban Hospital; CUHK: The Chinese University of Hong Kong, HONG KONG

## Abstract

*Pseudomonas aeruginosa*, an opportunistic pathogen associated with wound infections, resists many commonly available antibiotics. Its ability to form biofilm provides an additional trait to evade antibiotics. Biofilm-associated infections are difficult to treat, raising the need for alternative strategies. Thus, this research aimed to investigate the potential of bacteriophage to disrupt the biofilm produced by *P. aeruginosa* isolated from wound infections. Wound samples were collected aseptically, processed for the isolation of *P. aeruginosa*, and identified by standard microbiological methods. Antimicrobial susceptibility was determined by the Kirby-Bauer disc diffusion method. Bacteriophages were isolated using the double-layer agar method. Phenotypic assessment of biofilm formation by the isolates and its reduction by phages was conducted by the tissue culture plate assay. Out of 647 wound samples processed, 96 *P. aeruginosa* were isolated*.* Piperacillin/tazobactam was the most effective antibiotic, while doxycycline was the least effective. Among the total isolates, 86 (89.6%) were multidrug-resistant (MDR) and 69 (71.9%) were biofilm producers. Three different phages isolated from sewage demonstrated a high specificity to *P. aeruginosa*. Of these, phage vB_PaeP_PS2 lysed the highest number of isolates (22.9%), including 17 MDR and 21 biofilm-producing isolates. The biofilm reduction assay demonstrated that phage treatment significantly reduced biofilm formation, with vB_PaeP_PS2 achieving a 58% reduction after 6 h of treatment. In conclusion, this study highlights the high prevalence of biofilm-producing MDR *P. aeruginosa* in wound infections and, for the first time in Nepal, demonstrates the potential of locally isolated phages to lyse biofilm-forming MDR isolates and disrupt their biofilms.

## Introduction

Bacterial colonization of the wound leading to an infection is the most common complication impeding wound healing, which accounts for 70–80% of the mortality [1] and morbidity in patients, particularly in the developing countries, irrespective of the wound type [2]. Common bacterial pathogens associated with wound infection include *Staphylococcus aureus*, *Escherichia coli*, *Pseudomonas aeruginosa*, *Klebsiella pneumoniae*, *Streptococcus pyogenes*, *Proteus* species, *Streptococcus* species, and *Enterococcus* species [3].

Among these, *P. aeruginosa* is the most notorious due to its diverse virulence factors, such as lipopolysaccharide, flagella, and pili, secreted factors, exotoxin A, proteases, exoenzymes, phospholipase C, pyocyanin, alginate, DNase, and bacterial cell-to-cell interactions [4]. Besides, this organism is intrinsically resistant to numerous antibiotics, including anti-pseudomonal penicillins, aminoglycosides, and ciprofloxacin, owing to the low permeability of the outer membrane, expression of efflux pumps, porin alterations, beta-lactamases, aminoglycoside-modifying enzymes, etc. [5]. Due to these features, it is listed in the “Priority Pathogen” catalog published by WHO in 2024 [6]. Furthermore, biofilm formation is an added adaptive mechanism to protect from antibiotics [7].

Biofilm is a self-synthesized extracellular matrix composed of polysaccharides, lipids, proteins, and extracellular DNA, encasing the sessile cells to form bacterial communities [8]. It offers bacterial adherence and phenotypic resistance against antibiotics, host immune cells, and antibodies by limiting their diffusion within the biofilm matrix [9–11]. Owing to their complexity, biofilms are reported to be 10–1000 times more resistant than planktonic cells [12]. Thus, an alternative treatment strategy with an effective antibiofilm activity is required [13].

Bacteriophage, a natural predator of bacteria, has been considered a promising strategy against bacterial biofilms [14]. They are the most abundant biological entities in the environment, with population estimates of above 10^31^ viral particles. Therefore, they have been successfully isolated from diverse sources, including soil, water, sewage, humans, and animals [15]. Apart from their lytic property, they encode polysaccharide depolymerase enzymes, peptidoglycan hydrolase, and quorum quenchers that disrupt the complex structural components of biofilm [16,17].

Globally, the antibiofilm activity of phages against *P. aeruginosa* biofilms is well-documented. However, the antibiofilm potential of locally isolated phages has not yet been explored in Nepal. Therefore, this study aimed to evaluate the potential of bacteriophage as an anti-biofilm against *P. aeruginosa* associated with wound infection.

## Materials and methods

### Ethical approval

The Institutional Review Committee (IRC) of the Public Health Concern Trust, Nepal (PHECT- NEPAL), Kirtipur Hospital, Kathmandu, approved this study after submission of the application form with a research proposal, IRC application number 119–2023. Formal informed consent was waived by the Ethics Committee of PHECT-NEPAL, Kirtipur Hospital, Kathmandu, in accordance with national regulations and the Declaration of Helsinki. The waiver was granted due to the absence of direct contact or follow-up with patients and the minimal risk posed to participants.

### Study site, design, and period

A cross-sectional study was conducted from 07/11/2023–06/05/2024 at Kirtipur Hospital, Devdhoka, Kathmandu, and the Institute for Research in Science and Technology, Lalitpur.

### Study population and sample processing

The patients who visited Kirtipur Hospital with suspected wound infection were included. Clinical specimens like wound swabs, pus, and tissue were collected and transported immediately to the laboratory aseptically following the standard microbiological guidelines.

### Culture and identification of bacteria

The bacteria from the specimens were cultured on blood agar (BA), chocolate agar (CA), and MacConkey agar (MA) (HiMedia, India). The inoculated media were incubated aerobically at 37 °C for 24 h, except for CA plates, which were incubated with an additional 5–10% CO_2_ [18]. Identification was performed using standard microbiological techniques, including Gram staining and biochemical tests like catalase, oxidase, OF (oxidation-fermentation), indole, MR (methyl red)/VP (Voges-Proskauer), citrate utilization, urease, and TSIA (Triple Sugar Iron Agar) tests [19].

### Antibiotic Susceptibility Testing (AST)

The antimicrobial susceptibility test of the *P. aeruginosa* isolates was performed by the Kirby-Bauer disc diffusion method according to the Clinical and Laboratory Standards Institute guidelines (CLSI) [20]. *P. aeruginosa* ATCC 27853 was used as a positive control. Each isolate was tested against 10 standard antibiotic discs (HiMedia, India) from six different classes: cefepime (30 µg), ceftazidime (30 µg), amikacin (30 µg), gentamicin (10 µg), meropenem (10 µg), imipenem (30 µg), piperacillin/tazobactam (100/10 µg), ciprofloxacin (5 µg), levofloxacin (5 µg), and doxycycline (30 µg). Bacterial suspensions were adjusted to 0.5 McFarland before swabbing onto Mueller-Hinton agar. The inhibition zone diameter was measured after incubation for 18–24 h at 37 °C. Isolates were interpreted as sensitive, intermediate, and resistant based on CLSI [20]. Isolates non-susceptible to at least one antibiotic in three or more antimicrobial classes were considered multidrug resistant (MDR) [21].

### Biofilm assay

The tissue culture plate assay was used to estimate the biofilm as described elsewhere [22]. The isolated colony of *P. aeruginosa* was inoculated in 5 mL tryptic soy broth (TSB) with 1% glucose and incubated at 37 °C for 24 h. After incubation, the culture was diluted with fresh TSB medium (1:100) in a tissue culture plate (Tarsons, Korea) and incubated aerobically at 37 °C for 24 h. Then, the planktonic cells were pipetted out and the plate was washed 4 times with phosphate buffer solution (PBS, pH:7.2) and air-dried. Adherent biofilms were fixed with 2% sodium acetate for 5 min. The fixative was washed out using PBS, and biofilms were stained with 0.1% crystal violet (CV) for 15 min. The stain was aspirated, washed properly with PBS, and air-dried. The plate was solubilized with 200 µL of 95% ethanol for 30 min to detach the fixed cell from the well. The optical density (OD) of each well with solution was measured at 630 nm using an ELISA plate reader (HER 480, HT company, UK). *P. aeruginosa* ATCC 27853 and a sterile TSB + 1% glucose were used as positive and negative controls, respectively. All the experiments were performed in triplicate. The cut-off value (ODc) was defined as three standard deviations (SD) above the mean OD of the negative control: ODc = average OD of negative control + (3 × SD of negative control). Then, biofilm formation was interpreted as a non-biofilm producer (OD ≤ ODc), weak (ODc < OD ≤ 2 × ODc), moderate (2 × ODc < OD ≤ 4 × ODc), and strong biofilm producer (4 × ODc < OD) [23].

### Bacteriophage isolation and purification

Bacteriophages (in short phages) were isolated from sewage by double layer agar (DLA) method using *P. aeruginosa* ATCC 27853 as a host. The sewage sample was centrifuged at 4500 rpm for 10 min, and the supernatant was filtered with a 0.2 μm membrane filter (Omsons). One milliliter of filtrate was mixed with 0.5 mL of the exponential phase culture of host *P. aeruginosa* ATCC 27853, and 3 mL of molten soft agar (0.75%) was added to it. The mixture was overlaid over the solid bottom nutrient agar (NA) plate and incubated for 24 h at 37 °C. The plaques, which appeared as clear zones, were an indication of the presence of lytic bacteriophages.

For purification, a typical plaque was picked from the top agar layer using a sterile pipette tip and suspended in 1 mL SM buffer (50 mM Tris–HCl, 10 mM NaCl, and 8 mM MgSO4, pH 7.4), followed by vortexing. Phage was purified by the DLA method as described above. The purification process was performed repeatedly until morphologically identical plaques were obtained. The purified phages were resuspended in SM buffer and stored at 4 °C until further use [24].

### Host range determination and lytic activity of phages

The host range of the phages was determined by a spot assay with *S. aureus* (ATCC 29213 and 43300), *E. coli* (ATCC 25922 and 35218), *Klebsiella quasipneumoniae* (ATCC 700603), *E. faecalis* (ATCC 51299), and *P. aeruginosa* (ATCC 278583). The exponential phase culture of the host strain was mixed with 3 mL molten soft agar (0.75%) and overlaid on nutrient agar, and 5 µL of the phage (~10^7^ PFU/ml) was spotted on it, then incubated at 37 °C overnight. An appearance of clear zones was noted [25].

The lytic activity of phages was tested against the 96 clinical isolates of *P. aeruginosa* by the spot assay as described above.

### Biofilm reduction assay

To study the anti-biofilm activity of phages, 13 strong biofilm-forming *P. aeruginosa* isolates were selected based on OD reading. The biofilms formed on the wells of tissue culture plates were washed with 200 µL PBS. In each well a 200 µL of phage lysate (~10^8^ PFU/ml) was dispensed, except for the control well, which contained the biofilm of respective isolates without phage treatment (sterile TSB was added). The plate was incubated statically at 37 °C for 6 h, then the wells were washed with 200 µL PBS, followed by the biofilm fixation with 2% sodium acetate for 5 min. The fixative was washed out with PBS solution, and the wells were stained with 0.1% CV, as aforementioned. Then, 200 µL of 95% ethanol was added to each well, and OD_630_ was measured with an ELISA reader (HER 480, HT company, UK). All the assays were performed in triplicate [24].

### Data analysis

The biofilm reduction by phage was analyzed by one-way analysis of variance (ANOVA) to compare mean values of control and phage-treated groups. Then, Tukey’s Honestly Significant Difference (HSD) post hoc test was applied for pairwise comparisons. The experiments were performed in triplicate. Data are presented as mean ± standard deviation. These statistical analyses were performed in R Studio 2024.04.1 + 748 (R version 4.4.0). A *p* < 0.05 was considered statistically significant.

## Results

### Bacterial isolates from wound infection

**Fig 1:**
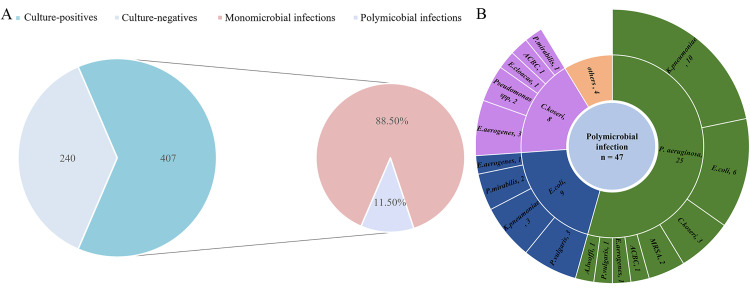
Culture positivity in wound samples. (A) Wound samples (n = 647) were processed for microbial growth at 37 °C on MacConkey agar, Chocolate agar, and Blood agar. The samples with growth of pathogens were considered culture-positives. The sample with growth of more than one bacterial pathogen was considered a polymicrobial infection. (B) Distribution of bacteria involved in polymicrobial wound infections.

Of the 647 wound samples, 407 (63%) were positive for bacterial growth. Among these culture positives, 47 (11.5%) showed polymicrobial growth (Fig 1A). *P. aeruginosa* and *K. pneumoniae* occurred together in 21.3% (10/47) of the wounds with polymicrobial infections. *P. aeruginosa* was the predominant species involved in the polymicrobial infection (Fig 1B).

**Fig 2:**
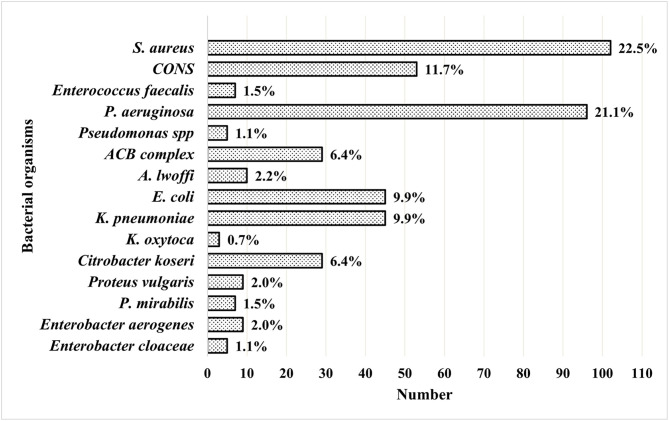
Frequency of bacterial isolates in wound infection. Bars represent the percentage frequency of each pathogen relative to the total isolates (n = 454).

A total of 454 bacterial isolates were identified from the culture-positive wound samples. Gram-negative bacteria were the most frequently isolated, representing 64.3% of the total isolates, while 35.7% were Gram-positive bacteria. Among the bacterial isolates, *S. aureus* (22.5%) was the predominant pathogen, followed by *P. aeruginosa* (21.1%), and *K. oxytoca,* with 0.7% being the least isolated bacterium (Fig 2).

### Antibiotic susceptibility of *P. aeruginosa*

**Fig 3:**
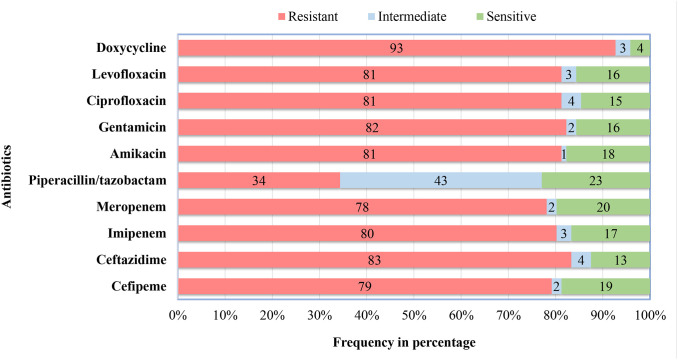
Antibiotic susceptibility of *P. aeruginosa* isolates. The stacked bar diagram shows the percentage of isolates that were resistant, intermediate, and sensitive to the different classes of antibiotics, as determined by the Kirby-Bauer disc diffusion test.

Out of 10 antibiotics tested, piperacillin/tazobactam was the most effective antibiotic since the highest number of *P. aeruginosa* isolates (23%) were sensitive to this drug. Meanwhile, doxycycline was the least effective drug, with only 4% of the isolates being sensitive (Fig 3). Besides, 86 (89.6%) of the total *P. aeruginosa* isolates were MDR, exhibiting resistance to more than three classes of antibiotics. Only two isolates were sensitive to all the antibiotics tested.

### Biofilm-producing *P. aeruginosa*

**Fig 4:**
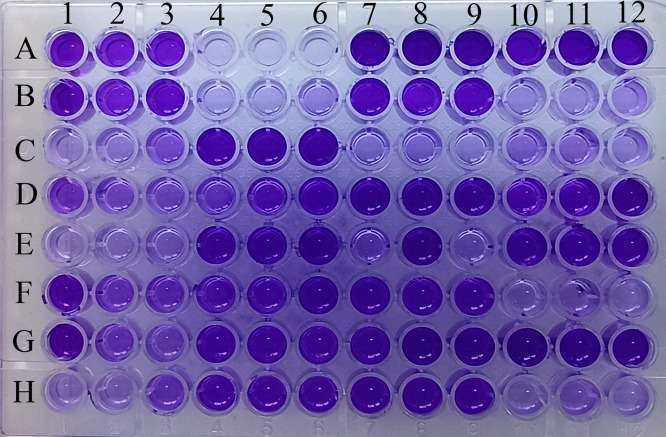
Biofilm assay. Clinical isolates, along with controls, were incubated in 96-well plates for 24 h at 37 °C, followed by crystal violet staining. The higher the biofilm formation, the greater the intensity of purple color. Wells A1- A3 represent positive control (P. aeruginosa ATCC 27853), A4-A6 are the negative control (TSB), and the clinical isolates were placed in the remaining wells in triplicate.

Biofilm formation was phenotypically observed in 69 (71.9%) *P. aeruginosa,* of which 19 isolates were strong, 20 were moderate, and 30 were weak biofilm producers (Fig 4).

### Host ranges and lytic activity of isolated phage

**Fig 5:**
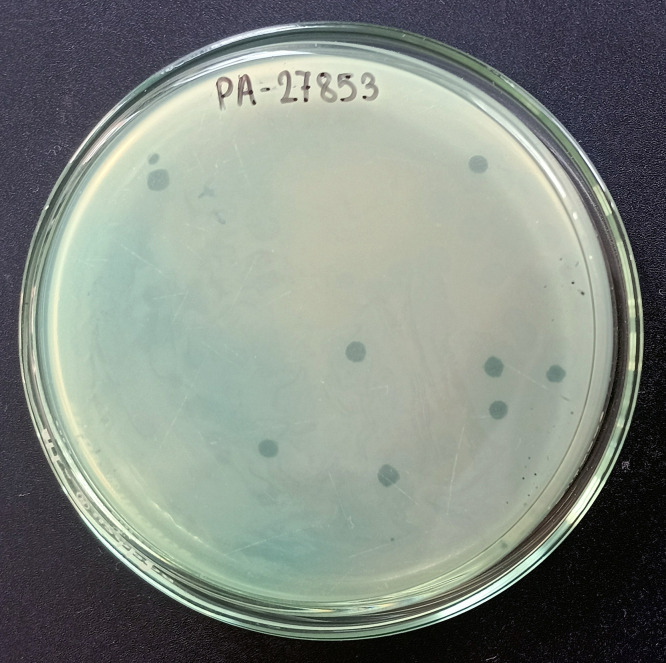
Phage isolation by the double-layer agar method. Clear zones of plaques formed by phage vB_PaeP_PS2 against P. aeruginosa ATCC 27853.

Sewage-contaminated river water samples were collected from the Bagmati River and the Icchumati River in Kathmandu. A total of three different phages were isolated from two different sewage-contaminated river water samples. Phages vB_PaeP_PS1 and vB_PaeP_PS2 produced clear, circular plaques of 2 mm diameter (Fig 5) while vB_PaeP_PS3 produced translucent, pinhead plaques.

All three phages were highly specific to *P. aeruginosa* ATCC 27853 and did not show lytic activity against non-*Pseudomonas* host species. When tested on the clinical *P. aeruginosa* isolates, the phages lysed 24 isolates (25%), which included 17 MDR and 21 biofilm formers. The phage vB_PaeP_PS2 lysed a broader range of clinical *P. aeruginosa* isolates, showing lytic activity against 22 isolates (22.9%). Among these 22 isolates, two were solely susceptible to phage vB_PaeP_PS2, while 12 isolates were susceptible to both phages vB_PaeP_PS1 and vB_PaeP_PS2. Four isolates were susceptible to vB_PaeP_PS2 and vB_PaeP_PS3. Likewise, 4 isolates were highly susceptible and were lysed by all three phages (S1 Fig).

### Biofilm reduction by phages

**Fig 6:**
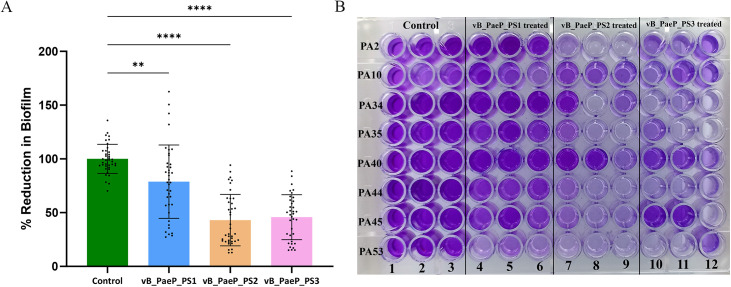
Overall biofilm reduction by phages vB_PaeP_PS1, vB_PaeP_PS2, and vB_PaeP_PS3. (A) Strong biofilm-producing P. aeruginosa isolates were grown in 96-well plates in triplicate. Control wells were incubated with sterile TSB, while treatment wells were incubated with the respective phages at 37 °C for 6 h. Following crystal violet staining, OD values were measured in an ELISA reader at OD_630_. The results were presented as mean ± SD. Bars indicate mean values, and error bars indicate standard deviation. The individual data points are represented as dots. (B) The intensity of the crystal violet stain reveals the biofilm mass. Wells in columns 1-3 are controls for the respective isolates; columns 4-6, 7-9, and 10-12 correspond to the treatment with phages vB_PaeP_PS1, vB_PaeP_PS2, and vB_PaeP_PS3, respectively.

Biofilm reduction assay was performed on the thirteen *P. aeruginosa* isolates selected among the strong biofilm formers. The biofilm was significantly reduced (**p* *< 0.05) when treated with phage for 6 h. Notably, phage vB_PaeP_PS2 showed the highest overall biofilm reduction, achieving 58% reduction across the tested isolates, compared to 22% and 45% for vB_PaeP_PS1 and vB_PaeP_PS3, respectively (Figs 6A and 6B).

**Fig 7:**
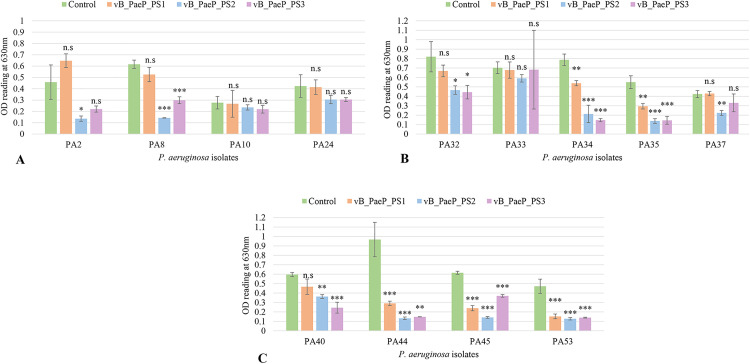
Effect of phages vB_PaeP_PS1, vB-PaeP_PS2, and vB_PaeP_PS3 against biofilm of different P. aeruginosa isolates. The experiment was carried out as three independent replicates and data were expressed as means ± SD, with p < 0.05 was considered significant. Asterisks represent significance of data where * = p < 0.05, ** = p < 0.01, and *** = p < 0.001. n.s indicates statistical non-significance of data.

The biofilm formed by the two clinical isolates, PA-45 and PA-53, was significantly reduced by all three phages (*p* < 0.001), whereas in isolates PA-10, PA-24, and PA-33, the reduction was not significant (*p* > 0.05). For the remaining isolates, at least one of the phages significantly reduced the biofilm (Fig 7). Overall, these results highlight vB_PaeP_PS2 as the most effective phage in reducing biofilms across the tested *P. aeruginosa* isolates.

## Discussion

Wound infections are prominent causes for mortality and morbidity, predominantly in developing countries [1]. Empirical antibiotic therapy remains the standard treatment approach; however, the rise of multidrug-resistant, biofilm-producing pathogens contributes to wound chronicity. While debridement is used to manage biofilms, it may necessitate limb amputation in severe cases, like diabetic foot ulcers [10]. These challenges underscore the need for novel strategies targeting both antimicrobial resistance and biofilm-associated persistence. Thus, this study aimed to investigate the potential of bacteriophages to disrupt the biofilm produced by *P. aeruginosa* isolated from the wound infection.

The current study found that 63% of the wounds were infected, which aligns with the culture-positive rates reported in previous similar studies conducted in Nepal [26,27]. However, one study on infected lesions from Nepal reported a lower bacterial growth rate [28]. In contrast, studies in African settings elucidated a higher culture positivity rate [29,30]. These variations may be attributed to various factors such as differences in the type of sample, prophylactic use of antibiotics, type of study population, study site, etc. [31].

Regarding the type of bacterial infection, wounds are predominantly associated with a single type of bacteria, which is consistent with other studies in Nepal [26,27,32]. However, polymicrobial infection of wounds, despite lower frequency, has also been detected, with *P. aeruginosa* most commonly involved. Polymicrobial infection has also been reported in Egypt [29]. The occurrence of monomicrobial or polymicrobial infection depends on the condition of the wound, microbial density, and wound care [29].

In this study, *S. aureus* was the predominant etiology followed by *P. aeruginosa*. This finding is also consistent with numerous studies on wound infections in Nepal, where *S. aureus* was the predominant organism, followed by other Gram-negative bacteria [27,33,34]. It is also congruent with the studies carried out in Bangladesh and Ethiopia, which reported *S. aureus* as the predominant isolate, followed by *P. aeruginosa* [31,35,34]. A high rate of occurrence of *S. aureus* in wound infection may be due to contamination of the wound from the endogenous source and unsanitary practices [29]. Meanwhile, *P. aeruginosa* is an opportunistic and nosocomial pathogen known for its diverse virulence factors, like biofilm formation, enzyme production, such as proteases, phospholipase C, etc., that mediate tissue damage and wound infection [31].

Piperacillin/tazobactam was the most effective antibiotic against *P. aeruginosa,* whereas doxycycline was the least effective in our study. This finding aligns with a study carried out in burn wounds in Nepal [36], while others have reported *P. aeruginosa* to be sensitive to the aforementioned antibiotics [26,32,35,37]. Similarly, this result is similar to the findings of other developing countries like Bangladesh and Iraq [34,38,39], while it is in contrast to a study carried out in Upper Egypt in 2023 [29]. Multidrug resistance in *P. aeruginosa* in this study was relatively higher than reported previously in Nepal [26,32,37], while it is similar to that reported from studies in Bangladesh [34,38]. Antibiotic resistance in *P. aeruginosa* is attributed to various intrinsic, acquired, and adaptive resistance mechanisms that evolved within the organism [5]. Besides that, prolonged and irrational use of antibiotics, prophylactic application in surgical cases, and use in poultry have contributed to the rapid development of antimicrobial resistance in bacteria [30].

In this study, the prevalence of biofilm-producing *P. aeruginosa* isolates from wound samples was 71.9%, which is relatively higher than previously reported in Nepal [36,40]. A similar trend of biofilm formation by *P. aeruginosa* in infected wounds has been described in a study from Ethiopia [31]. These findings reflect the burden of biofilm-forming pathogens in wound infections and highlight the need for rapid detection and effective wound management.

Conventional antibiotic treatment is insufficient to treat infections caused by MDR and biofilm-forming organisms. [41], creating urgency for novel strategies to combat these organisms. One of the strategies to address the problem is utilizing the phage possessing antibiofilm activity. Thus, we isolated phages against *P. aeruginosa* from sewage samples, which is considered a potential source for diverse phages. Sewage houses a plethora of microorganisms from human and animal origin, and its high nutrient content makes it likely to be a reservoir of a diverse population of phages [42]. The three isolated phages lysed 19.8% (17 of 86) of MDR *P. aeruginosa*, which is comparatively lower than reported in other recent studies on *Pseudomonas* phages [24,25,43]. Out of the three phages, phage vB_PaeP_PS2 showed lytic effect on the maximum number of isolates (n = 22) while phages vB_PaeP_PS1 and vB_PaeP_PS3 lysed 16 and 10 isolates, respectively. The variation in the lytic ability among the phages indicates that they are different from each other [42]. Overall, the phages in our study exhibited a narrower host range relative to previously reported *Pseudomonas* phages [44–46].

The biofilm reduction assay demonstrated a significant reduction in biofilm among clinical isolates. The phages disintegrated 22–58% of the biofilm after 6 h of treatment. Most clinical isolates showed a notable reduction in biofilm when treated with at least one type of phage; however, three isolates exhibited no significant biofilm-reduction with any of the phages tested. The reduction observed in this study is lower than that reported in some studies, which achieved up to 99% reduction of *P. aeruginosa* biofilm [17,44]. Meanwhile, it was comparable to findings from wound-derived *P. aeruginosa* biofilms [25]. Notably, the phages in this study significantly reduced biofilm within 6 h of phage exposure. The result is consistent with a 2022 study, in which phages significantly reduced biofilms of six clinical isolates after 6 h of treatment [43]. In contrast, another study reported that Phage treatment reduced biofilm mass by 80% at an MOI of 1 and by more than 95% at an MOI of 10 within 4 h, although biofilm regrowth occurred after 24 h [47]. Duration of phage-host contact is one of the factors to influence the phage efficacy, as studies have reported varying levels of biofilm reduction at different exposure times [44,48,49]. Other factors, like multiplicity of infection, incubation temperature, host strain, biofilm age, etc., also affect the rate of biofilm elimination [17,24,48,49]. Additionally, the composition and density of biofilm, the metabolic rate of the organism, and the development of phage-resistant phenotypes may also influence the outcomes [25].

This study was limited to the phenotypic evaluation of *P. aeruginosa* biofilm formation and its reduction by phages. The biochemical composition of the biofilm and the underlying mechanisms involved in biofilm reduction were unexplored. Further studies encompassing molecular analysis of the isolates and genomic characterization of phages would provide insightful information on the antibiofilm activities and help us understand the phage-mediated biofilm disruption.

## Conclusion

Since we found a high frequency of multidrug-resistant *P. aeruginosa* in wound infections, and also with the ability to form biofilm, it is very likely for a rise in difficulty to cure wound infections. Thus, the phages with the ability to lyse biofilm-forming MDR isolates and reduce the biofilm are the potential alternative for the control of life-threatening *P. aeruginosa* infections.

## Supporting information

S1 Fig Number of clinical isolates lysed by phages in single or combination. (TIF)
