# Supplementary figures and images for: Bacteriophage as an anti-biofilm agent against Pseudomonas aeruginosa from wound infection
Source: PLoS One. 2025 Oct 9;20(10):e0334139. doi: 10.1371/journal.pone.0334139 (PMC12510511; doi:10.1371/journal.pone.0334139)

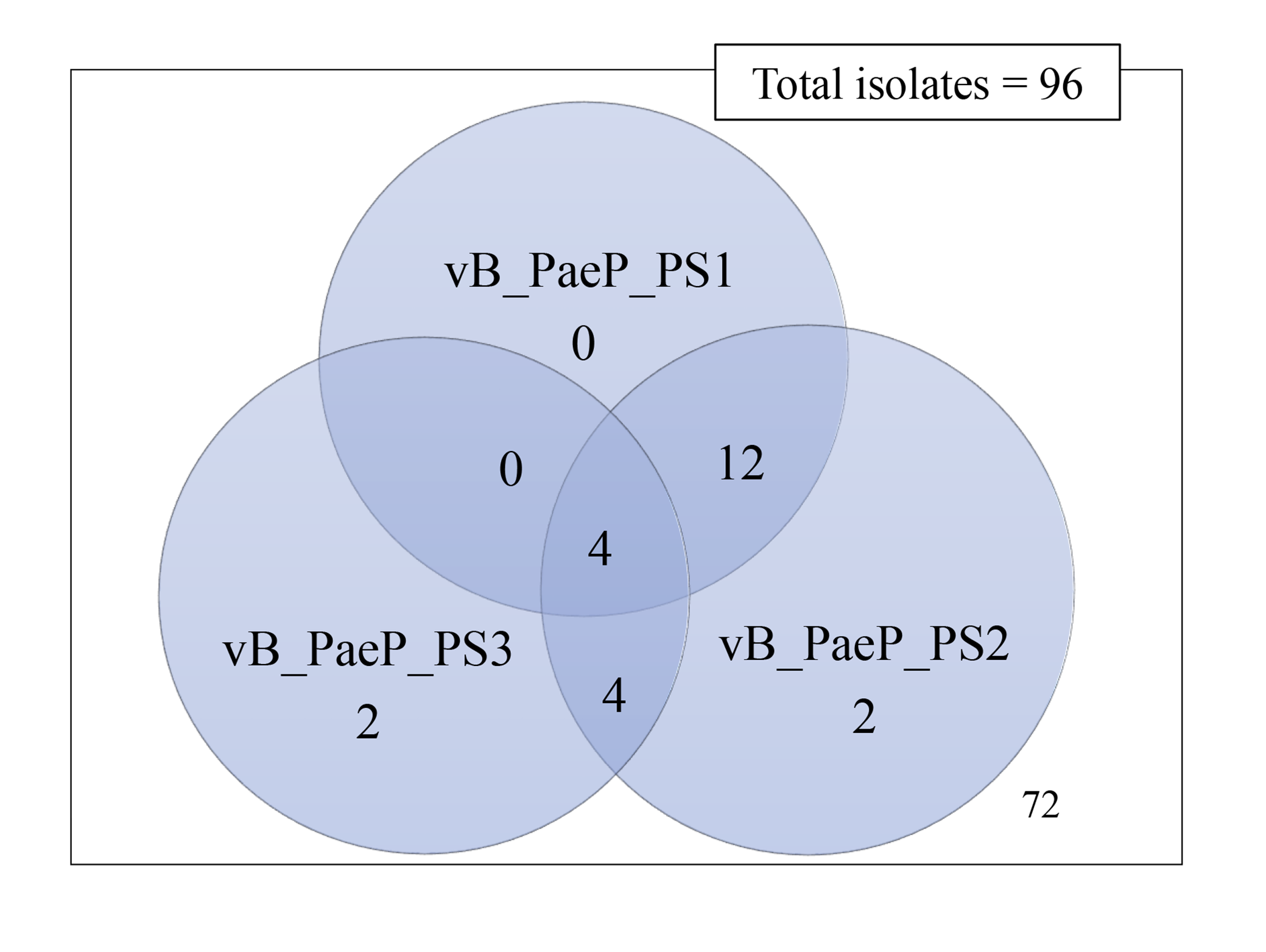

Supplement: S1 Fig — (TIF) [file pone.0334139.s001.tif]
